# Prussian-Blue-Nanozyme-Enhanced Simultaneous Immunochromatographic Control of Two Relevant Bacterial Pathogens in Milk

**DOI:** 10.3390/foods13193032

**Published:** 2024-09-24

**Authors:** Olga D. Hendrickson, Nadezhda A. Byzova, Boris B. Dzantiev, Anatoly V. Zherdev

**Affiliations:** A.N. Bach Institute of Biochemistry, Research Center of Biotechnology of the Russian Academy of Sciences, Leninsky Prospect 33, 119071 Moscow, Russia; odhendrick@gmail.com (O.D.H.); nbyzova@inbi.ras.ru (N.A.B.); dzantiev@inbi.ras.ru (B.B.D.)

**Keywords:** *Salmonella typhimurium*, *Listeria monocytogenes*, foodborne pathogens, multiplex immunochromatographic analysis, Prussian blue nanozyme, food safety

## Abstract

*Salmonella typhimurium* and *Listeria monocytogenes* are relevant foodborne bacterial pathogens which may cause serious intoxications and infectious diseases in humans. In this study, a sensitive immunochromatographic analysis (ICA) for the simultaneous detection of these two pathogens was developed. For this, test strips containing two test zones with specific monoclonal antibodies (MAb) against lipopolysaccharides of *S. typhimurium* and *L. monocytogenes* and one control zone with secondary antibodies were designed, and the double-assay conditions were optimized to ensure high analytical parameters. Prussian blue nanoparticles (PBNPs) were used as nanozyme labels and were conjugated with specific MAbs to perform a sandwich format of the ICA. Peroxidase-mimic properties of PBNPs allowed for the catalytic amplification of the colorimetric signal on test strips, enhancing the assay sensitivity. The limits of detection (LODs) of *Salmonella* and *Listeria* cells were 2 × 10^2^ and 7 × 10^3^ cells/mL, respectively. LODs were 100-fold less than those achieved due to the ICA based on the traditional gold label. The developed double ICA was approbated for the detection of bacteria in cow milk samples, which were processed by simple dilution by buffer before the assay. For *S. typhimurium* and *L. monocytogenes*, the recoveries from milk were 86.3 ± 9.8 and 118.2 ± 10.5% and correlated well with those estimated by the enzyme-linked immunosorbent assay as a reference method. The proposed approach was characterized by high specificity: no cross-reactivity with other bacteria strains was observed. The assay satisfies the requirements for rapid tests: a full cycle from sample acquisition to result assessment in less than half an hour. The developed ICA has a high application potential for the multiplex detection of other foodborne pathogens.

## 1. Introduction

Food security is one of the fundamental segments of public policy and a necessary condition for health, physical activity, high quality of life, and population longevity [1,2]. Ensuring food security is regularly on the agenda of any polity [3,4]. An important factor is controlling the undesired activity of pathogen microbes in food products [5]. Among other features of high-quality foodstuffs, their nutritional content, taste, and freshness can be noted.

Pathogenic bacteria are dangerous due to their ability to cause severe infectious diseases in animals and humans when they enter food and drinking water [6,7]. When exposed to a favorable environment, they multiply and can lead to serious negative consequences, such as livestock lethality or outbreaks of infectious diseases. The reasons for the proliferation of pathogenic microorganisms and their contamination of food products are poor hygiene of production and trade workers, improper food processing, transportation, and storage, and other factors [8].

*Salmonella* and *Listeria* are considered the most dangerous pathogenic bacteria [9,10]. Most often, they contaminate insufficiently thermally processed products: raw eggs, undercooked meat and poultry, and unpasteurized dairy products, and may cause intoxication and illness [11]. Especially abundantly, they multiply in products with liquid and semi-liquid consistency. Foremost, food prepared outside the home can be contaminated: in cafes, restaurants, fast food outlets, vending machines, delivery services, etc. The pathogenic bacterium *Salmonella typhimurium* causes salmonellosis, an infectious disease that most often occurs with injury to the gastrointestinal tract (provoking vomiting, profuse diarrhea, abdominal pain, and fever). In some cases, bacterial invasion may cause the development of a severe generalized process (with damage to other organs and systems) [12,13,14]. *Salmonella* are intracellular parasites. *Salmonella* infection is transmitted through the fecal–oral route. The infectious dose is 30–100 microorganisms or more and largely depends on the ingested food (products with high-fat content reduce the degree of bacterial invasion). After eating contaminated food and intestinal colonization, *Salmonella* penetrates the intestinal mucosa, causing inflammatory responses. In systemic diseases, *Salmonella* can disseminate through the bloodstream, accumulating in the lymph nodes and spleen [12,13,14]. *Salmonella* produces effectors that suppress cellular immune responses, which leads to the death of the host cell.

*Listeria monocytogenes* is also an intracellular parasite characterized by polymorphism of clinical manifestations and a high percentage of fatal outcomes. When entering a macroorganism, these bacteria induce the development of listeriosis caused by the production of protein pathogenicity factors that allow *Listeria* to penetrate eukaryotic cells and parasitize them [15,16]. The critical point in developing listeriosis is active invasion into enterocytes and subsequent crossing of the intestinal epithelial barrier. Listeriosis can occur either in the form of mild gastroenteritis or in the form of an extremely severe infection, especially in people with weakened immune systems—newborns, pregnant women, the elderly, and immunocompromised patients. In these cases, *Listeria* may provoke sepsis, meningitis, encephalitis, miscarriages, etc.

The current problem of antibiotic resistance only hinders the relief of these diseases and a favorable outcome for infected patients [17]. It should be noted that both *Salmonella* and *Listeria* can grow at low temperatures (2–8 °C), resulting in contamination of finished foodstuffs [18]. These microorganisms can contaminate frozen, smoked, or salted foods; cattle and poultry meat; hot smoked fish, sausages, butter, cheeses, confectionery creams, eggs, egg powder, and different dairy products. In this context, control of bacterial contamination in agro-industrial complexes, food production, livestock farms, food outlets, catering services, etc., is critically important to eliminate low-quality food products. The “gold standard” for identifying pathogenic microorganisms is the classic microbiological test in Petri dishes—microbial cultivation on nutrient media [19]. The main disadvantage of bacterial cultivation is the duration of microorganisms’ detection. The full cycle takes at least 3 days, which is unacceptable for the rapid production process in food enterprises and farms. Therefore, such advantages of the colony-culturing method as specificity (up to the genus of bacteria), accuracy, reliability, and sensitivity lose their attractiveness. Another promising way to detect pathogens is PCR testing aimed at repeated DNA amplification to produce a quantity sufficient for visual identification of the pathogen [20,21]. The advantage of the PCR method is the availability to analyze samples with minimum pathogen content and the avoidance of lengthy microbiological methods for long-growing bacteria. The PCR method is faster than the classical microbiological method and the detection of pathogenic microorganisms takes less time. However, such duration is also not always acceptable in the conditions of continuous food production. The main disadvantages of bacterial culturing and PCR are that they require the development of a full-fledged laboratory, the purchase of complex and expensive equipment, and the hiring of highly qualified personnel. The isothermal amplification and the classical enzyme-linked immunosorbent assay (ELISA) are also applied for bacteria detection [20].

Thus, for timely and effective control of food contamination, not only accurate and sensitive but also fast analytical methods are required, allowing for mass and rapid monitoring of many samples without the involvement of qualified operators and additional equipment. These methods include immunochromatographic analysis (ICA) implemented using test strips that are completely ready for use [22,23,24]. ICA is based on the reaction of an analyte with specific antibodies with the revealing of the immune interaction products using labels attached to one of the test components. Test strips are a multicomposite consisting of membrane carriers with pre-applied specific reagents. The testing process consists of dipping a test strip into the sample, incubating it for a short time (10–20 min), and visual (for qualitative analysis) or instrumental (for quantitative determination) assessing the assay results. Thus, the determination can be performed on-site at any point in the “from-farm-to-fork” chain.

Recent progress in immunochromatographic detection of the foodborne pathogen is summarized in some reviews [25,26,27]. *Salmonella* and *Listeria* ICA have been described in several studies [28,29,30,31,32]. In the majority of works, individual testing of these microorganisms has been developed. However, recently, a multiplex determination has become a promising trend in the immunoassay. The simultaneous detection of several relevant analytes increases testing productivity and reduces costs, time, reagents consumption, and the number of detected samples [33,34,35]. In the case of bacterial pathogens, multiparametric detection seems especially demanded because different pathogens can often induce similar symptoms. Hence, their differentiation is needed to take the necessary measures and prevent the spread of certain pathogens. Thus, both salmonellosis and listeriosis are often accompanied by gastroenteric disorders, and double analysis will help to distinguish these two pathogens. To our knowledge, the simultaneous determination of two or more pathogens using the ICA is presented mainly by complex analytical approaches. They are based on the combination of immunochromatography with recombinase polymerase amplification (RPA) [36,37,38] or surface-enhanced Raman scattering (SERS)-based detection [35,39,40]. Despite the excellent sensitivity (up to several cells in a milliliter of the detected sample) achieved in some of these works, the proposed approaches remain too complicated and expensive. Therefore, they cannot occupy a niche in rapid in-line tests for bacteria point-of-care control.

This work deals with the development of the first simple and fast multiparametric ICA of two relevant food pathogens—*Salmonella* and *Listeria*. As a label, a nanozyme based on the Prussian blue dye was used. Nanozymes are nanoparticles that imitate the catalytic properties of natural enzymes, most often, peroxidase [41,42,43,44,45]. Prussian blue nanoparticles (PBNPs) are a mixture of hexacyanoferrates and are also characterized by peroxidase-mimic properties [46,47,48].

The use of PBNPs can significantly increase the assay sensitivity in comparison to traditional gold labels. The mechanism of the enhancement is based on the nanozyme’s ability to catalyze substrate oxidation, which leads to the formation of a colored reaction product. This makes an additional contribution to the coloration of the analytical zones of the immunochromatographic test strip [30,49,50]. Increasing the ICA sensitivity is extremely important when testing food samples, the processing of which almost always requires multiple sample dilutions. Therefore, an approach has, for the first time, been proposed for the simultaneous immunochromatographic determination of *Salmonella* and *Listeria* with nanozyme enhancement. It should be noted that the application of nanozymes does not complicate the analytical protocol or make the assay dependent on the availability of extra equipment. The developed analysis was tested on cow milk samples.

## 2. Materials and Methods

### 2.1. Reagents and Materials

Potassium (II) hexacyanoferrate trihydrate (K_4_[Fe(CN)_6_] × 3H_2_O), citric acid, ferric chloride (III) hexahydrate (FeCl_3_ × 6H_2_O), gold (III) chloride hydrate (HAuCl_4_ × H_2_O), N-hydroxysuccinimide ester of biotin, sodium citrate, sodium azide, 1-ethyl-3-(3-dimethylaminopropyl)carbodiimide (EDC), N-hydroxysulfosuccinimide (sulfo-NHS), bovine serum albumin (BSA), sucrose, Tris, Tween-20, Triton X-100, 30% hydrogen peroxide, dimethyl sulfoxide (DMSO), and streptavidin conjugated with horseradish peroxidase (STR–HRP) were from Sigma-Aldrich (St. Louis, MO, USA). A chromogenic substrate kit for peroxidase activity based on 3,3′-diaminobenzidine (DAB) was purchased from Servicebio (Wuhan, China). A ready-to-use substrate solution based on 3,3′,5,5′-tetramethylbenzidine (TMB) was obtained from Immunotech (Moscow, Russia). The monoclonal antibodies (MAb) to lipopolysaccharide (LPS) of *S. typhimurium*, clone 1E6cc, and to LPS of *L. monocytogenes* (clones LZF7, LZH1, and LZG7) were from HyTest (Moscow, Russia). Goat anti-mouse immunoglobulins (GAMI) were from Arista Biologicals (Allentown, PA, USA). All other chemicals were purchased from Khimmed (Moscow, Russia); they were of analytical grade and were used without further purification. All solutions were prepared with ultrapure water with a resistivity of 18.2 MW (Millipore Corporation, Burlington, MA, USA).

The following bacterial strains were obtained from the State Collection of Pathogenic Microorganisms and Cell Cultures “GKPM–OBOLENSK” (Obolensk, Moscow region, Russia): *S. typhimurium*, *S.* Enteritidis 3-2, *S. paratyphi* A56, *S. virchov* 06, *S. anatum* 1120, *Escherichia coli* 0157:H7 ATCC51658, *Listeria monocytogenes* ATCC51658, *Yersinia enterocolitica* H-26-04, *Yersinia pseudotuberculosis* 4320, *Pseudomonas aeruginosa* ATCC27853, and *Francisella tularensis holarctica* 15.

A nitrocellulose working membrane (CNPC-SS12, having a 15 µm pore size), a glass fiber conjugate pad (PT-R7), a sample pad (GFB-R4), and an adsorbent pad (AP045) were purchased from Advanced Microdevices (Ambala Cantt, India). For the ELISA, transparent 96-well polystyrene microplates were purchased from Corning Costar (Tewksbury, MA, USA).

### 2.2. Biotinylation of MAb

Monoclonal antibodies to *L. monocytogenes* (clone LZG7) and *S. typhimurium* were biotinylated according to [51]. For this, 100 μM MAb solutions (200 μL) in 50 mM K-phosphate buffer with 100 mM NaCl with a pH 7.4 (PBS) were mixed with a 1 mM solution of N-hydroxysuccinimide ester of biotin (in DMSO) and stirred for 2 h at room temperature (RT). Excess unreacted low molecular weight reagents were removed by the dialysis against PBS.

### 2.3. ELISA

MAb to *S. typhimurium* or *L. monocytogenes* (clone LZF7) (1 μg/mL, 100 μL in PBS) were immobilized in the microplate wells overnight at 4 °C. Then, the microplate was washed four times with PBS containing 0.05% Triton X-100 (PBST). Next, solutions of *S. typhimurium* (1 × 10^7^–1 × 10^4^ cells/mL, 50 μL in PBST) or *L. monocytogenes* (2 × 10^8^–1.1 × 10^5^ cells/mL, 100 μL in PBST) were added to the wells and incubated for 1 h at 37 °C. After washing the microplate as described above, the corresponding biotinylated MAb (1 μg/mL, 100 μL in PBST) were added to the wells. The microplate was incubated for 1 h at 37 °C and again washed. After that, STR–HRP (1:5000 dilution, 100 μL in PBST) was added to the wells and incubated for 1 h at 37 °C. After washing, the activity of the enzyme label was determined. To do this, TMB-based substrate solution (100 μL) was added to the microplate wells and incubated for 10–15 min at RT. The reaction was terminated by adding 1 M sulfuric acid (50 μL) and the optical density (OD) was measured at 450 nm on a Zenyth 3100 microplate spectrophotometer (Anthos Labtec Instruments, Wals, Austria).

### 2.4. Synthesis of AuNPs and PBNPs

AuNPs with an average diameter of about 30 nm were prepared by the citrate synthesis [52]. Briefly, to 146.25 mL of ultra-pure water, 1.5 mL of a 1% solution of HAuCl_4_ was added, and the resulting mixture was heated to 100 °C. After that, 2.25 mL of a 1% solution of sodium citrate was added upon vigorous agitation. The mixture was kept boiling for 25 min.

The PBNPs were synthesized following the technique described in [50]. To each solution of FeCl_3_ and K_4_[Fe(CN)_6_] (1 mM, 20 mL), 98 mg of citric acid was added. The obtained mixtures were heated to 60 °C, quickly mixed, and incubated for 3 min. Then, the mixture was cooled to RT under stirring (~1.5 h). The obtained blue solution (16 mL) was centrifuged for 45 min at 20,000× *g*, and the supernatant was withdrawn. The precipitate was resuspended in 50 mM sodium phosphate buffer, pH 7.4, up to the initial volume. Both AuNPs and PBNPs were stored at 4 °C.

To characterize the dimensional parameters of labels, transmission electron microscopy (TEM) was implemented on a JEM-100C microscope (JEOL, Tokyo, Japan). Spectra were registered using Libra S50PC spectrophotometer (Biochrom, Cambridge, UK). Dynamic light scattering (DLS) measurements were performed on Zetasizer Nano ZS 90 (Malvern, UK).

### 2.5. Conjugation of Labels with Specific MAb

MAb (for *L. monocytogenes*—of the clone LZH1) were dialyzed against 10 mM Tris-HCl buffer, pH 9.0, before conjugation with AuNPs. The solution of AuNPs was adjusted to pH 9.2 by adding 0.2 M potassium carbonate. Then, the MAb (10 μg/mL) were added to the AuNPs (OD_520_ = 1) and stirred for 1 h at RT. Then, 10% BSA in ultrapure water was poured into the reaction mixture to 25% content and incubated for 15 min at RT. The resulting MAb–AuNPs conjugates were centrifuged for 15 min at 15,000× *g*. The sediments were dissolved in 0.01 M Tris-HCl, pH 9.0, containing 0.01% sodium azide, 1.0% sucrose, and 1.0% BSA (TBSA). Conjugates were kept at 4 °C for at least 3 months.

To conjugate with the MAbs (for *L. monocytogenes*—of the clone LZH1), PBNPs solution (4 mL), EDC (16 mg), and sulfo-NHS (32 mg) were mixed and incubated for 25 min at RT. The MAb were added to obtain concentrations of 10 μg/mL and stirred for 2 h at RT. After that, 10% BSA in PBS was added and incubated as described above. The obtained conjugates were washed by triple centrifugation at 14,500× *g* for 15 min, and the precipitates were resuspended in PBS. Finally, the MAb–PBNPs were resuspended in 400 µL of water solution of 1% BSA and 1% sucrose and kept at 4 °C. Conjugates were kept at 4 °C for at least 1 month.

### 2.6. Assembly of Test Strips

Several test strips were prepared: for AuNPs-based and PBNPs-based individual analyses and PBNPs-based double testing (see details in Table 1). For all of them, CNPC-SS12 nitrocellulose carrier (of 15 µm pore size) (Advanced Microdevices, Ambala Cantt, India) was used as a working membrane. As an adsorption pad, a sample pad, a conjugate pad, an AP045 membrane, a GFB-R4 membrane, and a PT-R7 fiberglass membrane (from the same manufacturer) were used. A test zone (T zone) and a control zone (C zone) were made using an Iso-Flow dispenser (Imagene Technology, Hanover, NH, USA) with a loading of 0.1 µL/mm. The application of the labeled MAb in TBSA containing 0.05% of Tween-20 on the conjugate pad proceeded manually, with a loading of 32 µL/cm. The membranes with immobilized reactants were kept overnight at RT and for 1.5 h at 37 °C. Then, different multicomposites were prepared (Table 1). The final test strips of 3.2 mm width were obtained by cutting the composites with a cutter (KinBio, Shanghai, China). After storage with silica gel in sealed packages for at least 2 months at RT, the analytical performance of the ICAs did not change.

### 2.7. Sample Preparation

Cow milk with 1.5% fat content was purchased from a local supermarket. It was 5-fold diluted by PBS containing 1% Tween-20 (PBSTw_1_) and used for the ICAs.

### 2.8. Individual ICAs with AuNPs

Test strips were positioned horizontally and standard solutions of *S. typhimurium* or *L. monocytogenes* in PBS (concentration range 3 × 10^7^–3 × 10^4^ cells/mL, 60 μL) were applied to the sample pads. After a 10 min incubation at room temperature, the test strips were scanned using a CanoScan 9000F scanner (Canon, Tochigi, Japan). The resulting digital images were processed using TotalLab TL120 1D v2009 software (Nonlinear Dynamics, Newcastle, UK) to measure the color intensity of the zones in relative units (RUs).

### 2.9. Individual ICAs with PBNPs

First, the test strips were processed by the blocking buffer. For this, test strips were put vertically in the PBSTw_1_ with 5% BSA (40 μL) and kept for 5 min. To the standard solutions of *Salmonella* or *Listeria* cells in PBSTw_1_ (2 × 10^8^–20 or 7 × 10^8^–73 cells/mL, respectively, 40 μL), the corresponding MAb–PBNPs conjugates (1.5 μL in both cases) were added and incubated for 3 min. Then, the test strips were put into these mixtures and incubated for 12 min. Then, the test strips were transferred to the PBSTw_1_ (40 μL) and incubated for 5 min. The final processing was performed as described above.

### 2.10. Double ICA

The test strips were processed using the blocking buffer as described above. Standard solutions of *Salmonella* or *Listeria* cells in PBSTw_1_ (2 × 10^8^–23 or 7.3 × 10^8^–73 cells/mL, respectively, 20 μL each) were mixed, and the MAb–PBNPs conjugates (1.5 μL in both cases) were added and incubated for 3 min. Further steps were the same as described above. For the amplification step, 1 μL of the DAB was applied to the T zone and incubated for 1.5–2 min.

### 2.11. Evaluation of the Immunoassay Results and Statistical Analysis

OriginPro 9.0 software from OriginLab (Northampton, MA, USA) was applied to obtain dependencies of OD_450_ (for the ELISA) or color intensity of the T zone (for the ICA) (y) versus *S. typhimurium* or *L. monocytogenes* concentrations (x). The analytical characteristics of the assays were estimated following [53]. In the ELISA, the instrumental LOD corresponded to 10% binding with the immobilized MAb. In the ICA, the visual LOD was estimated as the minimum cell concentration causing visible coloration in the T zone (i.e., at least 600–700 relative units, RU). All measurements were made in triplicate, and the means ± SE (standard errors) of bacterial cell concentrations were calculated. To evaluate the repeatability and reproducibility of ICA, intra-assay and inter-assay coefficients of variation (CV) were assessed (*n* = 6). For the analysis of milk, 5 repeats were made for each sample.

## 3. Results and Discussion

### 3.1. Preparation and Testing of the Assay Components

The MAb to *Salmonella* and *Listeria* were tested by ELISA in its sandwich format traditionally utilized for multivalent antigens. Here, specific antibodies immobilized on the solid phase and labeled ones in solution form a ternary complex with the detected antigen. In the implemented ELISA, biotinylated antibodies were used. The label introduction into a specific complex was performed using a streptavidin–peroxidase conjugate interacting with biotin. According to the obtained calibration curves (Figure S1), the LODs/working ranges of detectable concentrations were 7.2 × 10^4^/1.3 × 10^5^–4.3 × 10^6^ for *Salmonella* and 6.1 × 10^5^/2.0 × 10^6^–1.6 × 10^7^ cells/mL for *Listeria*. The confirmed immune reactivity between analytes and specific antibodies allowed for the development of the ICAs.

**Figure 1:**
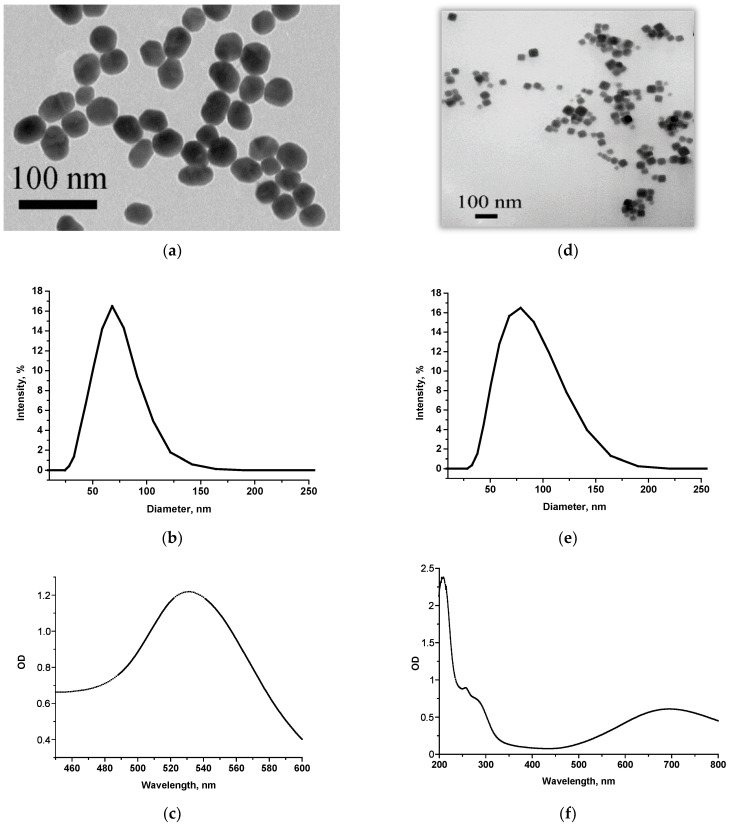
Characterization of markers carried out by TEM (**a**,**d**), DLS (**b**,**e**), and UV–vis spectroscopy (**c**,**f**) for AuNPs (**a**–**c**) and PBNPs (**d**–**f**).

First of all, ICA with the most commonly employed AuNPs-based labels was implemented as a comparison method. AuNPs were synthesized using a reduction in gold salt with sodium citrate [52]. Their characterization by size, shape, homogeneity, and aggregation was carried out by TEM. It was shown that the sample contained spherical non-aggregated objects with a diameter of 41.2 ± 2.3 nm (96 particles were processed) (Figure 1a). As the additional method of size assessment, DLS was applied. According to its results, the particle diameter was larger due to the hydration shell (with a maximum of ~70 nm) (Figure 1b). UV–vis spectrometry was used to confirm the compliance with typical spectra of nanoparticles of the same composition. The spectrum of AuNPs showed an absorption maximum at ~530 nm (Figure 1c).

Except for AuNPs, a catalytically active label based on PBNPs was applied to develop an enhanced ICA format. PBNPs are metal–organic frameworks, which were obtained by the reaction of ferric chloride with potassium hexacyanoferrate. This nanozyme has such properties as ease of synthesis and modification, structure controllability, high catalytic activity in the reaction of hydrogen peroxide reduction, etc. [42,46,47,48]. PBNPs are catalytically active over a wide pH range (2.5–7.4). The advantages of PBNPs compared to natural peroxidase converting the same chromogenic substrates are more stability and cheapness. All this makes PBNPs an attractive marker for ICA [30,49,50].

In this work, the surface of PBNPs was modified with carboxyl groups by citric acid to make conjugates with antibodies that contain amino groups. Except for TEM, PBNPs were also characterized by DLS and spectrophotometrically. According to the results obtained by TEM (Figure 1d), PBNPs had a cubic structure with an average size of 46.3 ± 11.0 nm (minimum value—28.4 nm, maximum value—76.2 nm, 76 objects were processed). According to DLS data, the particle diameter was about 80 nm (Figure 1e). The absorption peak in UV–vis spectra of PBNPs was ~700 nm due to the chromophore group of the pigment [54] (Figure 1f).

The resulting nano-dispersed labels were conjugated with specific MAb to *Salmonella* and *Listeria*. The conjugates were obtained by adsorption immobilization or by carbodiimide technique for AuNPs and PBNPs, respectively. In the former case, MAb concentration for conjugation was chosen following [51]. It assumes that MAb at the selected concentration should be sufficient to ensure the stability of AuNPs’ surface and prevent their aggregation. In this study, a MAb concentration of 10 μg/mL satisfied this requirement. In the case of the PBNPs, the carboxyl groups were first activated by NHS and EDC followed by the coupling of the MAb. Previous studies demonstrated that the optimal antibody concentration for conjugation with PBNPs of similar composition and size was 10 μg/mL. Higher MAb concentration leads to particle aggregation and less concentration—to the insufficient intensities of colorimetric signals in ICA [30,50]. Therefore, in this work, MAb with a concentration of 10 μg/mL were used to obtain all the labeled conjugates.

### 3.2. Individual ICAs with AuNPs

Firstly, ICA with a traditional nanogold label was developed. It was also carried out in a sandwich format, but unlike ELISA, the streptavidin–biotin module was not used, and the label was attached directly to the MAb. Two analytical zones were formed on the immunochromatographic working membrane—test (T) and control (C), where specific MAb to bacterium and anti-species antibodies (GAMI) were immobilized, respectively. The principle of immunochromatographic detection is the following: When a test strip is immersed in a sample, the movement of reagents with a liquid flow is started under the action of capillary forces. This is followed by immune interactions in the solution and on the membrane carriers. As a result, colored bands appear in the zones of the test strip; their existence/intensity is used to qualitatively/quantitatively estimate the assay results. In the sandwich ICA format, the analyte interacts both with MAb adsorbed in the T zone and labeled immunoglobulins. Therefore, the intensity of coloration directly relies on the analyte concentration. In the C zone, an excess of labeled MAb binds with GAMI. Typically, the colored C zone is used to judge the stored functionality of the test strip.

Two individual ICAs were developed to detect *Listeria* and *Salmonella* separately. For three available clones of anti-*Listeria* MAb, different combinations of immobilized MAb/labeled MAb were tested. The results displayed that the maximum sensitivity for *Listeria* detection was provided by the combination of immobilized clone LZF7 and labeled clone LZH1, which were subsequently used to develop test systems for *Listeria* cells (Table S1). The only MAb clone was used for the *Salmonella* test system; the use of antibodies of the same specificity is possible due to the existence of multiple repeating epitopes on the exterior of the cellular antigen.

**Figure 2:**
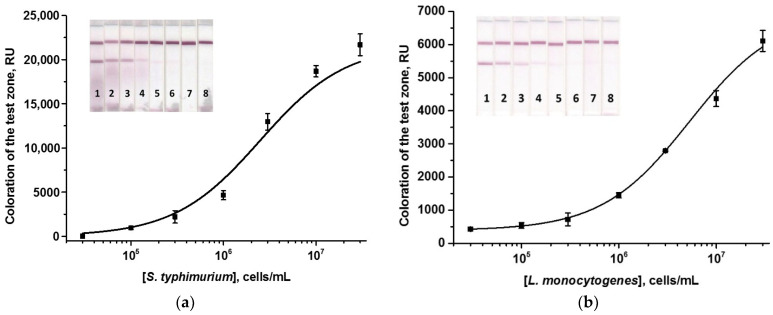
Calibration curves of *S. typhimurium* (**a**) and *L. monocytogenes* (**b**) in the AuNPs-based ICAs and the appearance of test strips. Concentrations of bacterial cells were 3 × 10^7^ (1), 1 × 10^7^ (2), 3 × 10^6^ (3), 1 × 10^6^ (4), 3 × 10^5^ (5), 1 × 10^5^ (6), 3 × 10^4^ (7), and 0 (8) cells/mL (n = 3).

To implement a monoparametric one-stage ICA, membrane multicomposites were used in the complete configuration, including the working membrane and 3 pads (Table 1). Here, and for all subsequent ICAs, conditions for bacteria detection were optimized to achieve minimum LODs. To do this, the concentration of immunoreagents and the time of the assay stage(s) were varied. All variable parameters are given in Table S2. In individual *Salmonella*/*Listeria* ICAs, the optimal parameters were the same: MAb concentrations in the T zones were 1 mg/mL, the GAMI concentrations in the C zones were 0.5 mg/mL, the concentrations of labeled MAb corresponded to OD_520_ = 6 in both cases, and the time of test strip incubation with the sample was 10 min. Calibration curves for *Salmonella* and *Listeria* cells are given in Figure 2. In the developed AuNPs-based test systems, the visual LODs of *S. typhimurium* and *L. monocytogenes* were 3 × 10^4^ and 3 × 10^5^ cells/mL, respectively; the assay duration was 10 min in both cases.

Antibody specificity was tested in AuNPs-based ICAs using a panel of pathogen cells, including several *Salmonella* species (*S.* Enteritidis 3-2, *S. paratyphi* A56, *S. virchov* 06, *S. anatum* 1120), as well as other microorganisms (*Escherichia coli* 0157:H7 ATCC51658, *Listeria monocytogenes* ATCC51658, *Yersinia enterocolitica* H-26-04, *Yersinia pseudotuberculosis* 4320, *Pseudomonas aeruginosa* ATCC27853, and *Francisella tularensis holarctica* 15). It was shown that the test system’s MAb were characterized by high specificity: the MAb interacted only with those antigens for which they were produced *(S. typhimurium* and *L. monocytogenes*, respectively). No cross-reactivity indicated the absence of T zone coloration (Figure S2) (signal intensity was not higher than 600–700 RU, which was invisible by the naked eye).

### 3.3. Individual ICAs with PBNPs

First, a standard colorimetric ICA was implemented with PBNPs, whose bright blue color provided well-visualized bands at test strips. However, the regimen for this ICA differed from that for AuNPs as labels. It is known that PBNPs often cause background signals due to non-specific adsorption of the nanoparticles on solid carriers and biopolymer molecules [50]. Therefore, additional stages had to be included in the analytical procedure: (i) blocking the membrane before the immune reaction and (ii) washing the membrane after the assay. In our previous work on the determination of myoglobin, the reaction media for PBNPs-based ICA were selected [30]. A phosphate buffer with increased detergent content (PBSTw_1_) and 5% BSA proved to be an optimal blocking medium. Pre-incubation of test strips with this medium eliminated the non-specific sorption of PBNPs on the working membrane and, accordingly, the development of a background signal.

For the same reason, all interactions were carried out in PBSTw1. In contrast to AuNPs-based ICA, antibody immobilization was also carried out in a special medium—phosphate buffer with 0.1% sodium azide, 0.25% sucrose, and 0.25% BSA [30]. It was not necessary to rinse the test strips after this ICA format. However, for unification with the enhanced PBNPs-based ICA (see below) and correct comparison of results for both assays, this stage was included in the final analytical protocol.

The test systems were optimized in many respects—test strip configuration, reagents’ concentrations, and duration of all stages (Table S2). The test strip composition was changed compared to AuNPs-based ICA: strips were cut to the lower edge of the working membrane because PBNPs tended to jam on both fiberglass and paper pads. Therefore, the MAb–PBNPs conjugate was added to the tested sample and pre-incubated to interact with the pathogen, and then the test strips were dipped into this mixture. Shortened tests had such advantages as reducing the sample volume (down to 40 µL), the total consumption of reagents, and the time required for reagents passage along the membrane. Thanks to the latter, the shorter incubation times of cut strips were needed. Although the assay was not a one-step, the total assay duration was 22 min, including 5 min blocking, 12 min interaction, and 5 min strip washing (assuming membrane blocking and pre-incubation were performed in parallel).

**Figure 3:**
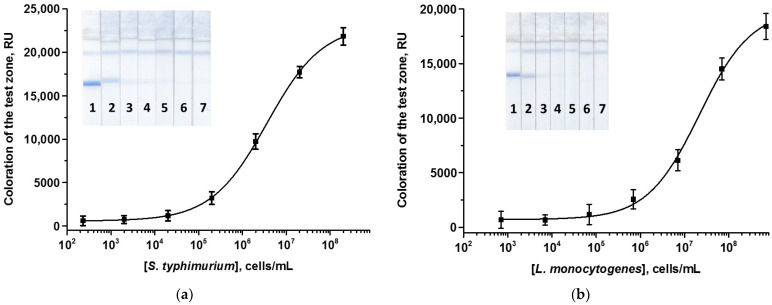
Calibration curves of *S. typhimurium* (**a**) and *L. monocytogenes* (**b**) in the ordinary ICAs using PBNPs and the appearance of test strips. Concentrations of *Salmonella*/*Listeria* were 2/7 × 10^8^ (1), 2/7 × 10^7^ (2), 2/7 × 10^6^ (3), 2/7 × 10^5^ (4), 2/7 × 10^4^ (5), 2/7 × 10^3^ (6), and 2/7 × 10^2^ (7) cells/mL (n = 3).

In optimized individual PBNPs-based ICA of *Salmonella*/*Listeria*, the concentrations of the immobilized Mab and GAMI and the volumes of the added MAb–PBNPs conjugates were the same: 2.5 and 0.5 mg/mL, and 1.5 μL (Table S2). Further increase in the concentration of labeled and/or immobilized antibodies increased the signal intensity (as expected for a sandwich ICA format). This, however, contributed to the development of the background at the zero point (in the absence of cells in the sample). Calibration curves for the detection of the pathogens are presented in Figure 3. The LODs were 2 × 10^4^ cells/mL for *S. typhimurium* and 7 × 10^5^ cells/mL of *L. monocytogenes*, being comparable with those for the AuNPs-based colorimetric ICAs.

### 3.4. Individual Enhanced ICAs with PBNPs

**Figure 4:**
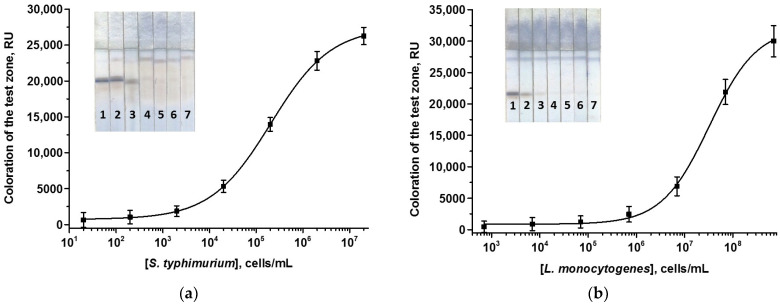
Calibration curves of *S. typhimurium* (**a**) and *L. monocytogenes* (**b**) in the enhanced ICAs using PBNPs and the appearance of test strips. Concentrations of *Salmonella*/*Listeria* cells were 2 × 10^7^/7 × 10^8^ (1), 2 × 10^6^/7 × 10^7^ (2), 2 × 10^5^/7 × 10^6^ (3), 2 × 10^4^/7 × 10^5^ (4), 2 × 10^3^/7 × 10^4^ (5), 2 × 10^2^/7 × 10^3^ (6), and 20/700 (7) cells/mL (n = 3).

For the enhanced ICAs, a PBNPs-based nanozyme was used. Peroxidase-like catalytic properties of PBNPs enable the oxidation of various peroxidase substrates. Enhanced ICA with PBNPs was carried out in the same manner as the assay without enhancement, except for an extra stage of catalytic signal amplification. A ready-to-use substrate mixture based on DAB was added after the main analytical procedure strictly to the T zone. The duration of the catalytic step was optimized to guarantee color development on the one hand and to avoid non-specific background on the other hand. Both demands were fulfilled by quick incubation of the test strips with DAB (1.5–2 min). All other parameters of the assays were the same in ordinary and enhanced ICAs. The resulting calibration curves for the enhanced ICA are presented in Figure 4. As can be seen, catalytic enhancement ensures a 100-fold lowering of LODs, namely 2 × 10^2^ and 7 × 10^3^ cells/mL for *S. typhimurium* and *L. monocytogenes*, respectively. The assay time, including the catalytic phase, was 24 min.

### 3.5. DoubleEnhanced ICA with PBNPs

Finally, a double ICA for the simultaneous detection of both pathogens was created. *Salmonella* and *Listeria* test systems were coupled into one strip. It should be noted that as a rule, multiparametric detection is not a crude hybrid of two test systems for one test strip, and it is essential to select conditions for the coexisting interactions of many components of several parallel immunochemical and lateral processes. Optimization of the dual test system began with inspecting the mutual impact of immunoreagents—assessing the occurrence of non-specific interactions when antibodies of diverse specificities are immobilized, and two labeled antibodies and standard dilutions of two analytes are combined into one system. For this purpose, the ICA was performed in several variants, including non-specific ones (Table 2).

One T zone coloration was visualized only in options (1) and (2), and two zones became blue in option (9) (Figure S2), that is, only in the case of specific interactions. The lack of colored T zones when implementing options (3)–(8) indicated the absence of reliable cross-influence of the reagents. The found regularities permitted a double detection without additional manipulations to eliminate non-specific interactions.

**Figure 5:**
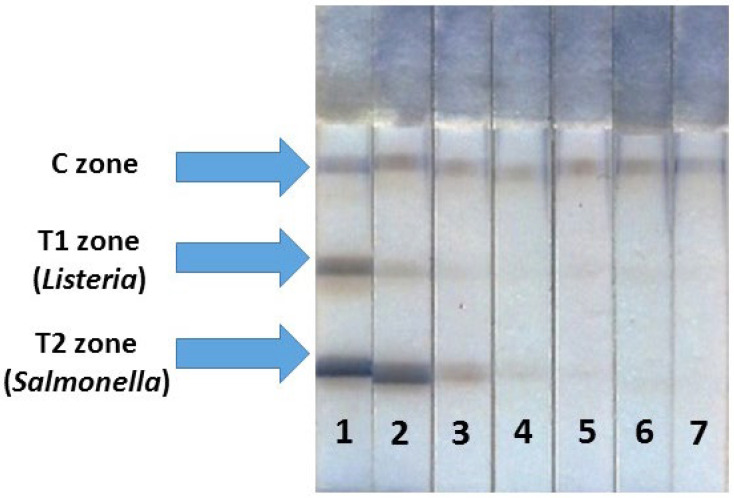
Appearance of test strips after the enhanced ICA of *S. typhimurium* and *L. monocytogenes* with PBNPs. Concentrations of *Salmonella*/*Listeria* cells were 2 × 10^7^/7 × 10^8^ (1), 2 × 10^6^/7 × 10^7^ (2), 2 × 10^5^/7 × 10^6^ (3), 2 × 10^4^/7 × 10^5^ (4), 2 × 10^3^/7 × 10^4^ (5), 2 × 10^2^/7 × 10^3^ (6), and 20/700 (7) cells/mL, respectively.

The number of T zones in multiplex assay corresponds to the number of analytes, so, in our case, two T zones were formed. The order of the T zone arrangement has to be selected when performing double detection because, in multiple ICAs, LODs can be altered depending on the locations of the binding zone on the test strips [55]. Two modes were tested: C zone → T1 zone (detection of *Listeria*) → T2 zone (detection of *Salmonella*) and C zone → T1 zone (detection of *Salmonella*) → T2 zone (detection of *Listeria*). It was found that the location of T zones generally did not affect the parameters of the double test system—the LODs and analytical signals did not change remarkably. For the double test system, the following location of the zones was chosen: C zone → T1 zone (detection of *Listeria*) → T2 zone (detection of *Salmonella*). The conditions of the double ICA were similar to those selected during the optimization of two monoparametric assays except for the GAMI concentration in the C zone: it was 2-fold increased because of double consumption of anti-species antibodies for binding to two labeled conjugates. The images of the test strip after the enhanced ICA are presented in Figure 5. The duration of all stages was kept as in individual tests. Hence, the detection of both analytes also took the same 22 min (plus 2 min for the catalytic amplification).

### 3.6. Detection of Bacteria in Milk

Milk was chosen as a foodstuff of interest because it is considered a healthy and nutritious product in the diet of grown-ups, children, and infants, often susceptible to contamination by foodborne pollutants. Cow milk with a fat content of 1.5% was bought at a local supermarket. As a rule, ICAs do not require complicated and careful sample preparation of analyzed samples, as arbitration analytical strategies do [56,57]. Nevertheless, it is still necessary to establish sample pretreatment conditions, under which the influence of milk matrix will not affect the assay results. For some liquid food samples, sample preparation is often not required, and the analysis is carried out directly in the sample [58,59]. In the case of milk, it is more frequently unrealistic because milk is a complex foodstuff containing various proteins, carbohydrates, fats, and other high- and low-molecular compounds. However, the matrix effect can be reduced simply and quickly by diluting the milk with a buffer [30]. In this study, 2-, 5-, and 10-fold dilutions of milk samples by PBSTw_1_ were tried as sample preparation ways. The initial samples were spiked with *Salmonella* and *Listeria* cells, diluted in the abovementioned regimes, and analyzed using the enhanced ICA. It has been revealed that a 5-fold dilution of milk was enough to get rid of the matrix impact (Figure S3). When milk was diluted 2 times, the sample flow along the membranes was inhibited probably due to an increase in the sample viscosity (compared to 5-fold diluted milk) ensured by fat content. So, the liquid front proceeded poorly, PBNPs became stuck at the bottom of the working membrane, and the analytical signal fell significantly. Upon 5- and 10-fold dilution of milk, testing progress did not differ from that in the buffer. Therefore, the minimally allowed dilution (5-fold) was selected so as not to lose sensitivity.

Experiments to determine recoveries were carried out using milk solutions with pathogen cell concentrations approximately corresponding to the inflection point of the calibration curves. For *Salmonella* and *Listeria*, concentrations of 1 × 10^6^ and 5 × 10^7^ cells/g were selected. Milk was contaminated with cells, then 5-fold diluted with PBSTw_1_, and the pathogen was detected using the enhanced ICA. The obtained recoveries (see Table 3) were 86.3 ± 9.8% and 118.2 ± 10.5% for *S. typhimurium* and *L. monocytogenes*, respectively. The same samples were analyzed by the ELISA as a reference method. As can be noticed (Table 3), the ELISA and ICA results are in acceptable agreement, which guarantees the correctness of the developed ICA.

### 3.7. Comparison with Other Studies

To confirm the relevance of our work and the significance of the results obtained, data on ICA of *Salmonella* and *Listeria* were assessed. The literature presents several studies on the individual immunochromatographic detection of *Salmonella* and *Listeria* using common gold markers [29,31] in various food matrices. Thus, Silva et al. (2024) developed a sandwich AuNPs-based lateral flow immunoassay for *Salmonella* detection in chicken, black pepper, milk, chocolate, and egg samples [29]. The ICA had an LOD of 10^3^ CFU/mL providing results in 15 min after incubation of the test strip with the sample. Mahari et al. (2023) designed an immunochromatographic test system for *Salmonella* detection using antibodies conjugated with AuNPs and smartphone-based detection [60]. The assay allowed for the determination of *S.* Gallinarum, *S. pullorum*, and *S.* Enteritidis in spiked fecal, meat, and milk samples with LODs in the range of 10^2^–10^4^ CFU/mL within 10 min. Wu et al. (2021) detected *S. typhimurium* using an AuNPs-based immunochromatographic test strip with a sensitivity of 4 × 10^5^ CFU/mL [61]. After 6–7 h of incubation, *S. typhimurium*, *S. paratyphi B*, and *S. enterica* could be detected in chicken, with an LOD as low as 1 CFU/mL. In a study by Lopes-Luz et al. (2023), antigenic targets of *L. monocytogenes* (proteins Internalins A and B that are involved in non-phagocytic cell invasion) were detected using anti-Internalin antibodies conjugated with AuNPs [62]. The test system could detect *L. monocytogenes* in the cell culture and milk with LODs of 5.9 × 10^3^ and 1 × 10^5^ CFU/mL, respectively.

Nanozyme labels of different compositions were also used for monoparametric analysis of *Salmonella* and *Listeria* [30,63,64,65]. In these works, the LODs for pathogens were lower than those in AuNPs-based ICAs and varied in the diapason from several tens to hundreds of thousands of cells/mL. Thus, in a study by Hu et al. (2023), using of iron oxide (Fe_3_O_4_) nanozymes allowed for achieving a LOD of 1 CFU/mL for *S*. typhimurium in juice, chicken, and lettuce leaves [64]. Hendrickson et al. (2023) developed an ICA of *S.* *typhimurium* in milk and chicken meat using an Au@Pt nanozyme label, which enabled reducing the LOD by two orders of magnitude compared to traditional AuNPs [30].

The presented new development, unlike its predecessors, combines not individual but multiplex ICA with nanozyme amplification. To our knowledge, there are no studies on lateral flow immunoassay for simultaneous determination of bacteria based on nanozyme enhancement of colorimetric signal. Multiplex determination of bacteria deals mainly with RPA-, PCR, or SERS-based ICAs. Although these approaches allow achieving some gain in the assay sensitivity (interestingly, in some cases, LODs are comparable with those in the common ICAs), they have some serious limitations for out-of-laboratory testing due to the necessity of complex equipment and special reagents (for example, primers or SERS tags) (see Section 1). Moreover, unlike the ICA developed in this study, complex analytical methods are much longer and cannot be considered a rapid analysis. The proposed assay is implemented without any extra equipment, and the signal amplification is initiated by simple substrate addition to the T zone of the test strip.

In our opinion, the only limitation of our study that may be noted is the introduction of the catalytic stage, which requires additional manipulations. However, this limitation can be neglected because the reaction with the substrate is not time-consuming (1.5–2 min) and only insignificantly reduces the testing productivity. This portable analysis can be completed within less than 30 min, including plain sample preparation fully meeting the requirements for rapidity. Thus, it can be assumed that this study represents the first simultaneous rapid and sensitive immunochromatographic detection of two bacterial pathogens combined with nanozyme enhancement.

## 4. Conclusions

In the present study, a double ICA has been developed for the simultaneous determination of *S. typhimurium* and *L. monocytogenes* as pathogens that may contaminate food and drinking water and generate foodborne diseases. The low LODs (2 × 10^2^ and 7 × 10^3^ cells/mL for *Salmonella* and *Listeria*, respectively) have been attained using Prussian blue nanoparticles as nanozyme labels. An additional catalytic stage increases the assay time by only 2 min and does not critically affect its rapidity. The developed ICA is sensitive, specific, rapid, and suitable for simultaneous point-of-care detection of two bacteria in milk. The implemented strategy can be extrapolated to different pathogens and contributes to rapid monitoring of foodstuffs’ safety and quality under non-laboratory conditions.

## Figures and Tables

**Table 1:** Composition of test strips used for the ICAs and the assay conditions.

Parameter	ICA of *Salmonella* with AuNPs	ICA of *Listeria* with AuNPs	ICA of *Salmonella* with PBNPs	ICA of *Listeria* with PBNPs	Double ICA with PBNPs
Absorption pad	Yes	Yes	Yes	Yes	Yes
Sample pad	Yes	Yes	No	No	No
Conjugate pad	Yes	Yes	No	No	No
Configuration of the test strip	Full	Full	Shortened *	Shortened	Shortened
The immobilization buffer	PBS	PBS	PBS+ **	PBS+	PBS+
MAb in the T zone(s), mg/mL	1	1 (clone LZF7)	2.5	2.5 (clone LZF7)	2.5 for both MAb
GAMI in the C zone, mg/mL	0.5	0.5	0.5	0.5	1
Labeled MAb immobilized on the conjugate pad, OD_520_	6	6 (clone LZH1)	No	No	No
Labeled MAb added into the tested sample, µL	No	No	1.5	1.5 (clone LZH1)	1.5 (both)
The sequence of the zones on the test strips (top down)	C zone → T zone	C zone → T zone	C zone → T zone	C zone → T zone	C zone → T1 zone (*Listeria* detection) → T2 zone (*Salmonella* detection)

* Test strips are cut to the bottom edge of the working membrane. ** PBS+ is PBS containing 0.1% sodium azide, 0.25% sucrose, and 0.25% BSA

**Table 2:** The results of the double ICA of *Salmonella* and *Listeria* performed in different regimes.

Option	Immobilized MAb	Detected Sample	Labeled Conjugate	T Zone Coloration
1	anti-*Salmonella*	*Salmonella* standard solution	anti-*Salmonella* MAb–PBNPs	T2
2	anti-*Listeria*	*Listeria* standard solution	anti-*Listeria* MAb–PBNPs	T1
3	anti-*Salmonella*	*Salmonella* standard solution	anti-*Listeria* MAb–PBNPs	no
4	anti-*Salmonella*	*Listeria* standard solution	anti-*Salmonella* MAb–PBNPs	no
5	anti-*Salmonella*	*Listeria* standard solution	anti-*Listeria* MAb–PBNPs	no
6	anti-*Listeria*	*Listeria* standard solution	anti-*Salmonella* MAb–PBNPs	no
7	anti-*Listeria*	*Salmonella* standard solution	anti-*Listeria* MAb–PBNPs	no
8	anti-*Listeria*	*Salmonella* standard solution	anti-*Salmonella* MAb–PBNPs	no
9	anti-*Salmonella* and anti-*Listeria*	*Salmonella* and *Listeria* standard solutions	anti-*Listeria* MAb–PBNPs and anti-*Salmonella* MAb–PBNPs	T1 and T2

**Table 3:** Recoveries of bacterial cells in ICA and ELISA of cow milk samples.

Parameter	ICA	ELISA
*S. typhimurium*		
Added, cells/g	1 × 10^6^	1 × 10^6^
Revealed, cells/g	1.2 × 10^6^ ± 1.1 × 10^5^	1.1 × 10^6^ ± 4.1 × 10^5^
Recovery ± SD *, %	118.2 ± 10.5	105.1 ± 4.1
*L. monocytogenes*		
Added, cells/g	5 × 10^7^	5 × 10^7^
Revealed, cells/g	4.3 × 10^7^ ± 4.9 × 10^6^	4.9 × 10^7^ ± 2.2 × 10^6^
Recovery ± SD, %	86.3 ± 9.8	97.9 ± 4.3

* Standard deviation, n = 3.

## Data Availability

The original contributions presented in the study are included in the article and Supplementary Materials; further inquiries can be directed to the corresponding author.

## Supplementary Materials

The following supporting information can be downloaded at: https://www.mdpi.com/article/10.3390/foods13193032/s1, Figure S1: Calibration curves of *S. typhimurium* (a) and *L. monocytogenes* (b) in the ELISAs; Figure S2: Study of non-specific interactions in the double ICA. T1 and T2 zones correspond to the immobilized anti-*Listeria* and anti-*Salmonella* MAb. The following combinations of the reagents were tested: anti-*Salmonella* MAb/*Salmonella* standard solution/anti-*Salmonella* MAb–PBNPs (1); anti-*Listeria* MAb/*Listeria* standard solution/anti-*Listeria* MAb–PBNPs (2); anti-*Salmonella* MAb/*Salmonella* standard solution/anti-*Listeria* MAb–PBNPs (3); anti-*Salmonella* MAb/*Listeria* standard solution/anti-*Salmonella* MAb–PBNPs (4); anti-*Salmonella* MAb/*Listeria* standard solution/anti-*Listeria* MAb–PBNPs (5); anti-*Listeria* MAb/*Listeria* standard solution/anti-*Salmonella* MAb–PBNPs (6); anti-*Listeria* MAb/*Salmonella* standard solution/anti-*Listeria* MAb–PBNPs (7); anti-*Listeria* MAb/*Salmonella* standard solution/anti-*Salmonella* MAb–PBNPs (8); anti-*Salmonella* and anti-*Listeria* MAb/*Salmonella* and *Listeria* standard solution/labeled anti-*Listeria* and anti-*Salmonella* MAb (9). Concentrations of both *Salmonella* and *Listeria* standard solutions were 1 × 10^8^ cells/mL; Figure S3: Images of test strips after the double ICA in PBST (a) and milk samples diluted 2 (b), 5 (c), and 10 (d) times. Concentration of both pathogens was 10^7^ cells/mL; Table S1: Parameters of the ICA of *L. monocytogenes* under different combinations of MAb clones in the test system (n = 3); Table S2: Parameters varied during optimization of the ICAs of *S. typhimurium* and *L. monocytogenes*.
