# Supplementary material for: Prussian-Blue-Nanozyme-Enhanced Simultaneous Immunochromatographic Control of Two Relevant Bacterial Pathogens in Milk
Source: Foods. 2024 Sep 24;13(19):3032. doi: 10.3390/foods13193032 (PMC11475848; doi:10.3390/foods13193032)
Supplement: Supplementary file 1 [file foods-13-03032-s001.zip › foods-3209290-supplementary.pdf]

Prussian blue nanozyme-enhanced simultaneous immunochromatographic control of two relevant bacterial pathogens in milk

Olga D. Hendrickson, Nadezhda A. Byzova, Boris B. Dzantiev, and Anatoly V. Zherdev*

A.N. Bach Institute of Biochemistry, Research Center of Biotechnology of the Russian Academy of Sciences, Leninsky prospect 33, 119071 Moscow, Russia; odhendrick@gmail.com (O.D.H.); nbyzova@inbi.ras.ru (N.A.B.); dzantiev@inbi.ras.ru (B.B.D.)

* Correspondence: zherdev@inbi.ras.ru (A.V.Z.); Tel.: +7-495-954-28-04

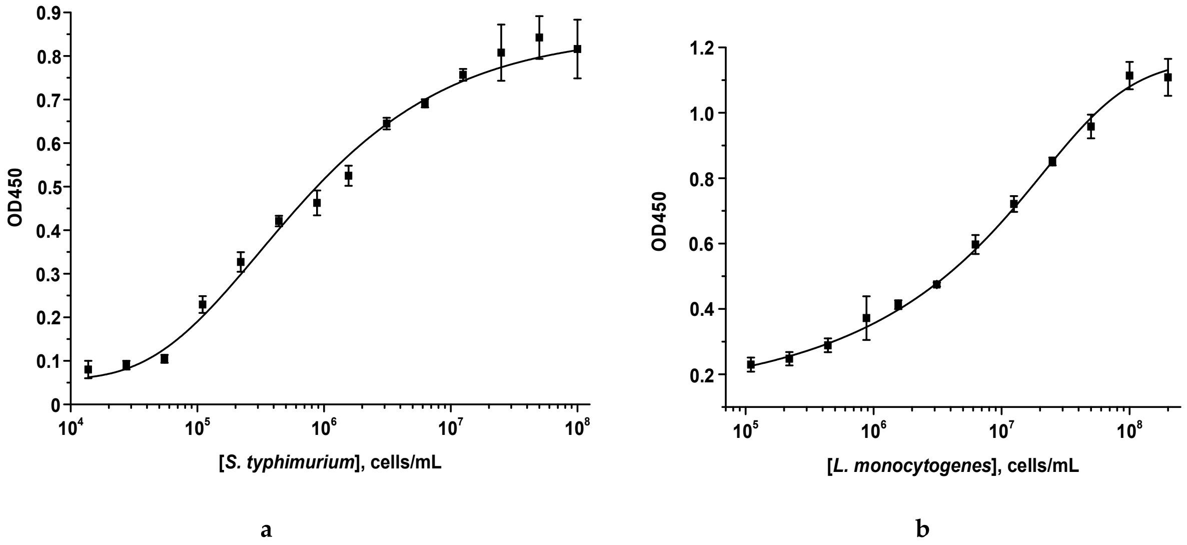

Figure S1. Calibration curves of *S. typhimurium* (a) and *L. monocytogenes* (b) in the ELISAs.

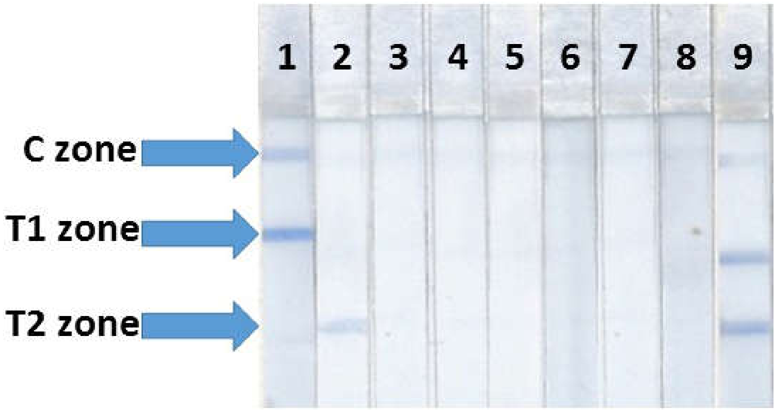

Figure S2. Study of non-specific interactions in the double ICA. T1 and T2 zones correspond to the immobilized anti-Listeria and anti-Salmonella MAb. The following combinations of the reagents were tested: anti-Salmonella MAb/Salmonella standard solution/anti-Salmonella MAb–PBNPs (1); anti-Listeria MAb/Listeria standard solution/anti-Listeria MAb–PBNPs (2); anti-Salmonella MAb/Salmonella standard solution/anti-Listeria MAb–PBNPs (3); anti-Salmonella MAb/Listeria standard solution/anti-Salmonella MAb–PBNPs (4); anti-Salmonella MAb/Listeria standard solution/anti-Listeria MAb–PBNPs (5); anti-Listeria MAb/Listeria standard solution/anti-Salmonella MAb–PBNPs (6); anti-Listeria MAb/Salmonella standard solution/anti-Listeria MAb–PBNPs (7); anti-Listeria MAb/Salmonella standard solution/anti-Salmonella MAb–PBNPs (8); anti-Salmonella and anti-Listeria MAb/Salmonella and Listeria standard solution/labeled anti-Listeria and anti-Salmonella MAb (9). Concentrations of both Salmonella and Listeria standard solutions were 1×10^8 cells/mL.

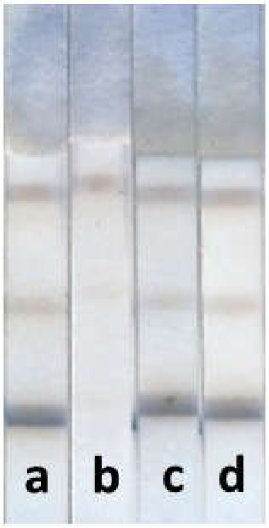

Figure S3. Images of test strips after the double ICA in PBST (a) and milk samples diluted 2 (b), 5 (c), and 10 (d) times. Concentration of both pathogens was 10^7 cells/mL.

Table S1. Parameters of the ICA of *L. monocytogenes* under different combinations of MAb clones in the test system (n = 3)

Combination of immobilized/labeled clones of MAb	LOD, cells/mL	Signal intensity, RU	Background, RU
LZF7/LZH1	3.1×10^5	~6100	380
LZF7/LZF7	n/i*	n/i	n/i
LZF7/LZG7	n/i	n/i	n/i
LZH1/LZH1	1.6×10^6	~3750	0
LZH1/LZF7	n/i	n/i	n/i
LZH1/LZG7	n/i	n/i	n/i
LZG7/LZH1	1.8×10^6	~5340	690
LZG7/LZF7	n/i	n/i	n/i
LZG7/LZG7	n/i	n/i	n/i

Table S2. Parameters varied during optimization of the ICAs of *S. typhimurium* and *L. monocytogenes*

Parameter	Varied diapason	Selected value
	Monoparametric AuNPs-based ICAs of *S. typhimurium*/*L. monocytogenes*	
Concentration of the immobilized MAb, mg/mL	0.25–2	1
Concentration of the immobilized GAMI, mg/mL	0.2–0.6	0.5
OD_{520} of the MAb–AuNPs conjugate	1–8	6
Assay duration, min		
	Monoparametric PBNPs-based ICAs of *S. typhimurium*/*L. monocytogenes* (in colorimetric and catalytic modes)	
Concentration of the immobilized MAb, mg/mL	0.25–2 for all	1/1
Concentration of the immobilized GAMI, mg/mL	0.2–0.6 for all	0.5/0.5
Volume of the added MAb–PBNPs conjugate, μL	1–5for all	1.5/1.5
Duration of membrane blocking, min	3–10 for all	5/5
Duration of pre-incubation of tested sample with labelled MAb	2–10 for all	3/3
Duration of incubation of test strips, min	10–15 for all	12/12
Duration of washing of test strips, min	3–10 for all	5/5
Volume of the added DAB, μL	1–25 for catalytic mode	1
Duration of the catalytic stage, min	1–5 for catalytic mode	2
	Multiparametric PBNPs-based ICA of *S. typhimurium* and *L. monocytogenes*	
Concentration of the immobilized MAb, mg/mL	1–3 for both	2.5/2.5
Concentration of the immobilized GAMI, mg/mL	0.5–1 for both	1/1
Volume of the added MAb–PBNPs conjugate, μL	1–5 both	1.5/1.5
Duration of membrane blocking, min	3–10	5
Duration of pre-incubation of tested sample with labelled Mab	2–10 both	3
Duration of incubation of test strips, min	10–15	12
Duration of washing of test strips, min	3–10	5
Volume of the added DAB, μL	1–25	1
Duration of the catalytic stage, min	1–5	2
